# A novel method for real-time adulteration identification of heated-honey processed Astragali radix using REIMS-iKnife and its regulatory effects on gut microbiota, SCFAs, and immune function in spleen-deficient rats

**DOI:** 10.3389/fphar.2026.1711584

**Published:** 2026-04-29

**Authors:** Pengpeng Liu, Yisheng Xu, Haoran Yu, Tianzhu Jia, Ji Shi, Yingyuan Lu, Pengfei Tu, Qian Cai

**Affiliations:** 1 Key Laboratory of Ministry of Education for TCM Viscera-State Theory and Applications, Liaoning University of Traditional Chinese Medicine, Shenyang, Liaoning, China; 2 School of Pharmacy, Liaoning University of Traditional Chinese Medicine, Dalian, Liaoning, China; 3 Waters Technology (Beijing) Co., Ltd., Beijing, China; 4 State Key Laboratory of Natural and Biomimetic Drugs, School of Pharmaceutical Sciences, Peking University, Beijing, China

**Keywords:** Astragali radix, heated honey processed astragali radix, intestinal flora, reims, SCFA, spleen deficiency

## Abstract

**Background:**

Astragali radix (AR) is a well-known traditional Chinese medicine (TCM). AR processed with heated honey (HAR) can enhance the effect in treating spleen deficiency. However, some people have used heated sugar as a substitute for heated honey in processing AR. The mechanism underlying the effect of HAR in ameliorating spleen deficiency has not been fully elucidated so far. Therefore, this study aimed to determine whether AR has been processed with heated honey or heated sugar. Subsequently, we compared the differential effects of AR and HAR on the composition of intestinal microbiota, short-chain fatty acid (SCFA) metabolism, and immune responses in spleen-deficient rats.

**Methods:**

Rapid evaporative ionization mass spectrometry (REIMS) was employed to detect AR processed with different types of heated honey, different types of heated sugar, and heated honey mixed with heated soft sugar in various proportions. 16S rRNA high-throughput sequencing was conducted to analyze the effects of AR and HAR on intestinal flora diversity in spleen-deficient rats. The regulatory effects of AR and HAR on SCFA metabolism in spleen-deficient rats were measured using headspace gas chromatography-mass spectrometry (HS-GC-MS). The effects of AR and HAR on specific and non-specific immunoreactivity in spleen-deficient rats were evaluated using enzyme-linked immunosorbent assay (ELISA). Furthermore, correlations were detected between different intestinal flora, short-chain fatty acids, and immune response factors.

**Results:**

We successfully identified AR processed with heated potpourri honey, linden honey, and locust flower honey as genuine heated honey processed products. Additionally, we achieved accurate identification of AR processed with heated soft sugar or granulated white sugar and determined the proportion of adulterated sugar in samples. Both AR and HAR increased the abundance of beneficial gut bacteria, reduced the abundance of pathogenic bacteria, upregulated the synthesis of propionic acid and butyric acid, and modulated the metabolism of isovaleric acid. Notably, the positive effect of HAR on propionic acid expressinon was stronger than that of AR. Meanwhile, both AR and HAR significantly modulated the expression levels of immune factors in serum and colon samples, with HAR exhibiting superior regulatory effects compared to AR.

**Conclusion:**

The procedure developed in this study can help rapidly and accurately determine whether sugar has been added during the heated honey processing of AR. The enhanced effect of HAR on spleen deficiency may be associated with its regulatory effect on the abundance of *Lactobacillus* and *Turicibacter* genera. Through this effect, it promotes the synthesis of propionic acid, upregulates anti-inflammatory factors, downregulates pro-inflammatory factors, and enhances the immunomodulatory effect.

## 1 Introduction

TCM must be processed to minimize its toxicity and increase its functional efficacy. Among the available methods (e.g., cutting, boiling, steaming, vinegar, salt-water, river sand stir-fry, rice-wine, and heated-honey), treating TCM products with heated honey is one of the most important methods. For the first time, the *Supplement to Prescriptions Worth a Thousand Gold Piece* from the Tang dynasty in 682 described honey as an adjuvant for TCM processing (Wang, 1998). However, nowadays, this process is often adulterated, which can affect the functional efficacy of TCM.

Since honey costs considerably more than granulated or soft sugar, some merchants substitute soft sugar, granulated white sugar, or their mixture with honey to produce ‘heated-honey’ for processing TCM. While this technique reduces production costs and increases profits, it also affects the functional efficacy of TCM. Heat can convert sucrose, the main ingredient of soft and granulated white sugars, into glucose and fructose (Ou et al., 2024). Glucose and fructose constitute approximately 70% of honey (Valverde et al., 2022). Therefore, it is difficult to accurately and quickly detect and identify heated honey processing decoction pieces produced from soft sugar, granulated white sugar or honey mixed with a portion of soft or granulated white sugar. Although it has been reported that the analysis of NIR spectroscopy and ^13^C/^12^C isotope ratios can identify adulterated honey containing glucose, fructose and fructose syrup, the pre-treatment process for sample analysis is complicated, time-consuming, and expensive (Downey et al., 2003; Tosun, 2013). However, this study utilized mass spectrometry (MS) technique to identify heated honey processing TCM adulterated with sugar, remove false samples, and save true samples, thereby ensuring processed product quality, functional safety, and efficacy.

Due to its rapidity, efficiency, and high sensitivity, MS has been widely employed in qualitative identification and quantitative determination of a number of complex samples, such as metabolites (Dinore and Farooqui, 2022; Wu et al., 2024), environmental pollutants (Mary Celin et al., 2023), drug residues (Desmarchelier et al., 2021; Pietruk et al., 2021), and illegal food additives and pesticides (Harischandra et al., 2021; Liao et al., 2022; Qi et al., 2023). Similarly, advanced MS can obtain accurate molecular quality and rich structural information in studying TCM, which is a complex system. Our strategy aims to realize accurate identification and recognition of samples. Especially, MS offers unique advantages and greatly improves analytical efficiency in the research of some TCM that are easily adulterated or forged. With the vigorous development of MS, emerging technologies, such as ion mobility mass spectrometry (IMS), mass spectrometry imaging (MSI), and multi-reflecting time-of-flight (MRT), are continuously emerging (Feider et al., 2019; Lacalle-Bergeron et al., 2023; Verenchikov et al., 2024). REIMS is also one of the many advanced types of MS technologies. Since its introduction in 2013, REIMS has been widely used in analyzing clinical samples, food authenticity or quality determination, chemical material identification, and plant and microbiological research (Shen et al., 2022; Song, Zhao, et al., 2021; Wang et al., 2023). This technique involves a high current electrosurgical knife (the “iKnife”, Supplementary Figure S1) that rapidly heats and cuts a sample to produce gas-phase ions on its surface. These gas-phase ions can enter the mass spectrometer inlet through a venturi pump, impact a heated impactor, and de-cluster. Differential markers can be identified using multivariate statistical analysis.

AR is derived from the root of *Astragalus membranaceus* (Fisch.) Bge. var. *mongholicus* (Bge.) Hsiao or *A. membranaceus*. AR is a Chinese botanical drug with the same origin as medicine and healthy food. It has a long history of consumption and replenishes qi and promotes yang. HAR can enhance the ability to tonify qi, strengthen the spleen, and is a good candidate for treating spleen deficiency syndrome (Cisema, 2020). The first record of HAR appeared in *Essence of the Silver Sea* published in the Tang dynasty. Currently, HAR is the main processed AR product in clinical practice. According to Chinese medicine theory, the spleen is responsible for transportation and is the source of qi and blood production. It is also closely associated with immune function and nutritional status. The abundance of probiotics, such as *Bifidobacterium*, *Lactobacillus*, and *Enterobacteriaceae*, is significantly lower in the ileum and cecum of animals with spleen deficiency. Conversely, the abundance of pathogenic bacteria, such as *Bacteroidetes*, *Enterococcus* and *Escherichia coli*, is significantly higher in these animals, which impairs the homeostasis of intestinal flora (Zhang et al., 2023). Imbalanced intestinal flora homeostasis has been shown to compromise the physiological roles of intestinal flora on the intestinal mucosa. These roles encompass defending against pathogens, stimulating the maturation of the immune organs, and activating the immune response. Consequently, imbalanced intestinal flora homeostasis compromises the immune function of the intestinal tract (Zhou et al., 2020). Enterobacteria can metabolize undigested carbohydrates into biologically active SCFAs via pyruvate, succinate, lactate, and acetyl coenzyme A (Oliphant and Allen-Vercoe, 2019), thereby compromising the function of the host immune system (Rooks and Garrett, 2016).

Therefore, for the first time, this study employed REIMS to rapidly and accurately differentiate AR processed with heated honey from that adulterated with heated sugar. We also explored the regulatory effects of AR and HAR on the intestinal flora, immune activity, and SCFAs in spleen-deficient rats, thereby providing a scientific basis for enhancing the efficacy and rational application of HAR.

## 2 Materials and methods

### 2.1 Materials

ARs were identified as *Astragalus membranaceus* (Fisch.) Bge. var. *mongholicus* (Bge.) Hsiao by Professor Ji Shi (Liaoning University of Traditional Chinese Medicine), and further confirmation was conducted based on microscopic characteristics. All voucher samples were deposited in the authors’ laboratory (Liaoning University of Traditional Chinese Medicine, Dalian, China).

Ultrapure water was provided using a Milli-Q system (18.2 MΩ, Millipore, MA, United States). Methanol, leucine enkephalin (LC-MS grade), formic acid, and acetonitrile were purchased from Thermo Fisher Scientific (Fair Lawn, NJ, United States). Three Chinese honeys and two sugars were examined, including locust flower honey (Beijing Hundred Flowers Bee Products Technology Development Co., Ltd. (Beijing), linden honey (Shanghai Guanshengyuan Bee Products Co., Ltd. (Shanghai)), potpourri honey (Beijing Tongrentang Bee Products Co., Ltd. (Beijing)), granulated white sugar (Beijing Sugar, Tobacco and Wine Group Co., Ltd. (Beijing)) and soft sugar (Nanjing Ganzhiyuan Co., Ltd. (Nanjing)). Acetic acid, propionic acid, butyric acid, isobutyric acid, valeric acid, isovaleric acid, hexanoic acid, internal standard, and 4-methylpentanoic acid were purchased from Shanghai Yuanye Biotechnology Co. Interleukin 10 (IL-10), interleukin-18 (IL-18), interleukin-1β (IL-1β), γ-interferon (IFN-γ), tumor necrosis factor-α (TNF-α), immunoglobulin A (IgA), and immunoglobulin G (IgG) enzyme immunoassay kits were purchased from Shanghai Kexing Company Limited (batch numbers: F3068-A, F3067-A, F3066-A F3071-A, F3070-A, F2923-A, F3074-A, F3056-A, F2938-A, and F2940-A). Rat D-Xylose content detection kit was purchased from Beijing Solebo Technology Co., Ltd., and Buzhongyiqi pills (water pills) were purchased from the Pharmaceutical Factory of Beijing Tongrentang Science and Technology Development Co., Ltd. Seventy healthy male SD rats (200 ± 10 g) were purchased from Liaoning Changsheng Biotechnology Co., Ltd. (Liaoning, China; Certificate No. SCXK (Liao) 2020-0001). The rats were acclimatized for 1 week under controlled conditions (24 °C ± 1 °C, 30%–50% relative humidity, and 12/12 h light/dark cycles), and had free access to chow and water.

### 2.2 Sample preparation

AR: The root of *Astragalus membranaceus* was cleaned to remove impurities, washed, dampened, cut into thick slices and dried.

HAR preparation was conducted based on the *Processing of TCM*, edited by Jia, (2008). The processing of HAR is briefly described in the following Section: 1) 25% of the weight of raw AR slices was weighed and diluted with hot water in a container (heated-honey-water ratio of 3:2); 2) next, raw AR slices were added and mixed evenly, smothered, and moisturized until almost 100% infiltrated with heated honey; 3) the saturated slices were then stir-fried at 120 °C–130 °C for 5 min. Heated soft sugar-processed AR (SSAR) was generated in the same way, but heated soft sugar was used instead of heated honey to prepare SSAR. Other samples were similarly prepared with different excipients (e.g., granulated white sugar, linden honey, potpourri honey) or proportions (honey mixed with a portion of soft or granulated white sugar).

**FIGURE 1:**
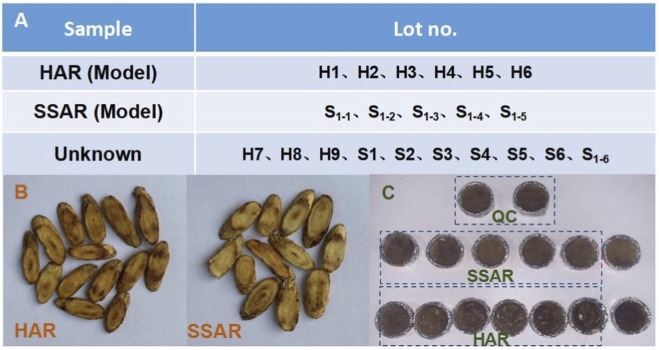
REIMS experimental samples. **(A)** Sample information. **(B)** HAR and SSAR. **(C)** State of the sample after dissolution with water.

REIMS study samples were ground into powder and then passed through a 50-mesh sieve. Next, 1.0 g powder of each sample was placed in a round cap wrapped with aluminum foil, and dissolved in 1.5 mL of ultrapure water (Figure 1). For quality control (QC), an appropriate amount of test HAR powder was weighed and mixed for each sample to appraise the stability and reproducibility of the REIMS method. Seven batches of AR processed with heated honey and six batches of AR processed with heated soft sugar were modeled for identification. Ten batches of processed products were examined. Three batches of AR were processed by mixing different proportions of heated soft sugar in heated honey.

Simple parching AR: AR was parched for 6 min at 130 °C-150 °C, and then cooled down.

Raw rhubarb decoction: An appropriate amount of raw rhubarb was weighed, and decocted with 10-fold, 8-fold, and 6-fold volumes of water sequentially. The decoction was filtered, and the filtrates were combined, concentrated to 1 g of crude drug per milliliter (1 g/mL) and stored at 4 °C for later use.

AR decoction: An appropriate amount of AR was weighed, and 10, 8, and 6 times the amount of water was added for decoction. After filtering, we combined the filtrate, concentrated it to 0.54 g mL^-1^, and stored it at 4 °C for subsequent use. The decoction of HAR and simple parching of AR were done using the same operation.

Positive drug decoction: Buzhongyiqi pills were dissolved in water and diluted to a concentration of 0.1 g/mL.

### 2.3 REIMS analysis

REIMS analysis was conducted using a Waters Xevo G2-XS quadrupole time-of-flight mass spectrometer with a REIMS ion source (Waters, Milford, MA) following the reported conditions. Briefly (Chen et al., 2024), a monopolar iKnife (Model PS01-63H, Hangzhou Medstar Technology Co, Ltd, Jiaxing City, China) was connected to a 3-m-long, 1-cm-diameter ultra-flexible tube (evacuation/vent line) to apply a localized high-frequency current to the sample surface. An Erbe VIO 50 C generator (Erbe Medical United Kingdom Ltd, Leeds, United Kingdom) was utilized for electrosurgical dissection of samples. All samples were burnt to produce an aerosol. The aerosol was then drawn into a PTFE tube connected to a venturi gas injection pump to the REIMS ion source. Optimal iKnife ionization conditions were applied, including 50 V (cone voltage), 1 s (cutting time), 1 cm (cutting length), and 60 V (heater bias voltage). Each sample was cut 10 times sequentially, with 2–3 s between two consecutive cuts. MS was conducted in negative ion mode with a mass range of 50–1200 m/z. Real-time calibration was conducted using a leucine enkephalin solution (200 pg/μL) formulated from 0.1% formic acid: 50% acetonitrile/water (v/v) and introduced continuously at a speed of 2 μL/min.

### 2.4 Spleen deficiency model preparation and treatment

Sixty rats were randomly selected, fasted on a single day, and fed 75 g/kg/day on a double day, with free access to water. A lead with 10% of the rats’ body weight was tied to the root of their tails, and the rats swam daily in 28 °C-32 °C water until exhaustion. The criterion for exhaustion was not reaching the surface for 7 s after immersion in water. After swimming, each rat was gavaged with 2.0 mL/100 g rhubarb decoction for 15 days. The body weight and anal temperature of the rats were recorded every 3 days. The model rats were monitored daily for the presence of diarrhea, hypophagia, lethargy, squinting, dark fur, and reduced activity, while rats in the blank group behaved normally. After 15 days of model establishment, three rats were randomly gavaged with 5% d-xylose solution. After 1 h, blood samples were collected from the orbital region to measure d-xylose content and investigate whether the spleen deficiency model was constructed successfully (Li et al., 2024; Wang et al., 2025).

Following successful model establishment, the rats were randomly assigned to one of the following groups: the model (MC) group; the positive drug (PC) group; the heated honey (HC) group; the simple parching AR (FC) group; the AR (RH) group; and the HAR (HH) group. The PC group received a dose of 1.62 g/kg/day, while the HC, FC, RH and HH groups received a dose of 8.1 g/kg/day. Rats in the NC and MC groups received an equal daily dose of saline. All rats had free access to food and water, and each treatment was administered for 15 days.

### 2.5 Immune organ index analysis

After the last dose, rats were fasted for 12 h but were allowed to drink water. Each group of rats was subsequently anesthetized via intraperitoneal injection of 20% urethane solution (0.4 mL/100 g). Then, the spleen and thymus of rats were dissected, dehydrated using filter paper, and weighed precisely. The thymic and splenic indexes were calculated using the following formulas: Spleen index (mg/g) = spleen weight (mg)/body weight (g) × 100. Thymus index (mg/g) = thymus weight (mg)/body weight (g) × 100.

### 2.6 Measurement of serum and colon immune indicators

Following anesthesia, blood samples were obtained from the abdominal aorta and collected into anticoagulant-free EP tubes to prepare serum samples. Then, colon tissues were harvested. The serum and tissue levels of relevant immune indicators, such as IL-6, IFN-γ, IL-18, IL-1β, TNF-α, IgA, IgG, IL-10, IL-4, and IL-2, were measured using ELISA kits.

### 2.7 Histopathological examination

Colonic tissues were cut into 1.0 cm × 1.0 cm pieces and fixed in 4% paraformaldehyde. After fixation, the samples were dehydrated with alcohol, soaked in xylene, and embedded in paraffin. Next, the samples were cut into 0.5 μm-thick slices, stained with hematoxylin and eosin (H&E), and observed under a light microscope.

### 2.8 Intestinal flora analysis

16S ribosomal RNA (rRNA) analysis of cecal samples was conducted by Suzhou Panomike Biomedical Technology Co, LTD (Jiangsu, China). After extracting total genomic DNA from feces, the microbial 16S rRNA V3-V4 region was amplified. High-throughput sequencing was performed using the Illumina MiSeq PE250 platform at the Realbio Genomics Institute (Shanghai, China). Chimeric sequences were filtered, and sequences with more than 95% similarity were regarded as the same operational taxonomic units (OTUs) as QIIME2. Alpha and beta diversity analyses were conducted using QIIME2.

### 2.9 SCFAs analysis

In each group, the intestinal contents of rats (100 mg) were precisely weighed and placed in a 20 mL headspace injection vial. Thereafter, 50 μL of 0.2% H_3_PO_4_ solution containing 4-methylpentanoic acid internal standard solution (0.668 mg/mL) was added, sealed rapidly, and employed as a test article for online measurement (Wu et al., 2023). For headspace injection, heating temperature of the sample bottle of the headspace injector was 80 °C; heating temperature of the dosing loop was 140 °C; heating temperature of the transfer line was 160 °C; GC cycle time was 30 min; heating time was 20 min; equilibrium time set to 10 min; pressurization time was set to 0.15 min; and injection time was set to 0.5 min (Rohde et al., 2022). Chromatographic conditions were as follows: the chromatographic column was an Agilent DB-WAX (DB-1MS) capillary column (30 m × 0.25 mm × 0.25 μm); the injection mode: no shunt; the temperature of the injection port was 250 °C; the temperature of the ion source was set to 230 °C; the temperature of the transmission line was set to 250 °C, the temperature of the quadrupole was set to 150 °C; and the programmed temperature increase was 60 °C. The temperature increased to 120 °C with a speed of 30 °C/min, and the temperature increased to 20 °C with a speed of 5 °C/min. The starting temperature was 60 °C, which subsequently increased to 120 °C at a speed of 30 °C/min. Next, the temperature increased to 140 °C at a speed of 5 °C/min, and this temperature was maintained for 1 min. Thereafter, the temperature increased to 150 °C at a speed of 10 °C/min, and this temperature was maintained for 1 min. In the next step, the temperature increased to 160 °C at a speed of 5 °C/min, and this temperature was maintained for 1 min. Finally, the temperature was increased to 230 °C by 35 °C/min, and that temperature was maintained for 5 min. Helium was used as the carrier gas at a flow rate of 1.0 mL/min (Kim et al., 2022). Mass spectrometry conditions were as follows: electron bombardment ion source (EI), electron energy: 70 eV; solvent delay: 4.5 min; scanning in full scan and selected ion monitoring (SIM) modes; scanning range: 30-200 m/z. Mass spectrometry parameters of the seven SCFAs and the internal standard are summarized in Table 1.

**TABLE 1:** Mass spectrometric parameters of seven SCFAs and the internal standard.

No	Chemical compound	Retention time (min)	Quantitative ion (m/z)	Qualitative ion (m/z)
1	Acetic acid	4.84	64.0	42.0,44.0
2	Propanoic acid	5.78	72.0	48.0,55.0
3	Isobutyric acid	6.14	73.0	45.0,84.0
4	Butyric acid	7.00	60.0	72.0,450
5	Isovaleric acid	7.67	60.0	88.0,45.0
6	Valeric acid	8.76	71.0	62.0,44.0
7	4-Methylpentanoic acid	10.01	57.0	71.0,83.0
8	Hexanoic acid	10.81	62.0	42.0,72.0

### 2.10 Correlation analysis

Spearman’s rank correlation coefficient analysis is widely employed as a non-parametric statistical method to explore multivariate correlations between potential biomarkers of intestinal microorganisms, parameters of the host immune system, and the concentrations of SCFAs. Correlation coefficients (*r*) and *P*-values were utilized to describe the degree of correlation between potential microbial biomarkers, immune indicators, and SCFAs. The correlation coefficients ranged from 0.5050 (maximum positive correlation) to −0.5100 (maximum anti-correlation), with 0 suggesting no correlation. In the cross-correlation heat map, blue indicates positive correlations, while red represents negative correlations. In this study, two sets of data with |*r*| < 0.6 and *P* < 0.01 were deemed significantly correlated.

### 2.11 Data processing and analysis

REIMS data acquisition and peak extraction were conducted using MassLynx 4.2 (Waters, Milford, United States). The mass spectral signals of 10 peaks from each sample were extracted for modeling. Data were analyzed using LiveID 1.2 software (Waters, Milford, MA). Parameters for “the cross-validation of the model by leaving 20% out samples” in LiveID were as follows: 1 linear discriminant; a binning resolution of 0.1 m/z; 5 standard deviations; 3 principal component analysis (PCA) components; and a mass range of 50–1200 m/z. Default Leu-Enk settings were used for demo data.

Data from animal experiments are presented as mean ± SD. One-way analysis of variance (ANOVA) and Tukey’s *post hoc* test was employed for multiple comparisons. GraphPad Prism 8.0 (San Diego, CA, United States) was employed to compare data from animal experiments. Letter notation (a, b, c, d) was used for multiple comparisons. Values with the same letter indicate no significant difference, whereas values with different letters indicate a significant difference. Differences were considered statistically significant when *p* < 0.05. Heat maps were plotted using Origin software (Hampleton, Massachusetts, United States). Redundancy analysis (RDA) was conducted using Canoco 5 software (Ithaca, NY, United States).

## 3 Results

### 3.1 Model construction and validation and multivariate statistical analysis

The radiofrequency current was applied directly to samples through the iKnife. The obtained samples were rapidly heated to produce an aerosol, which then released gas-phase ions into the vapor. The vapor was drawn through the sample transport tube into the mass spectrometer for analysis. Based on the MS information of the six batches of HAR and the five of SSAR, a real-time identification model was built using LiveID software to distinguish HAR (in honey) from SSAR (in sugar). This model was validated through “stratified 5-fold” validation, and the samples were used to establish each model divided into five groups (on average). Among them, 20% of model samples were used to evaluate the accuracy of model prediction. This process was conducted by collecting data from heated honey processed samples for classification prediction. Subsequently, data were collected from heated sugar processed samples for classification prediction and five cross-validations. Model accuracy and generalizability were determined based on the output report, the total number of correct and incorrect classifications, and the number of outliers. Of 103 mass spectra, there were zero outliers, and 100% of samples were correctly identified. This finding indicates that the model was stable, reliable, and statistically significant. Therefore, the model can be adopted to predict whether a test sample is HAR or SSAR.

**FIGURE 2:**
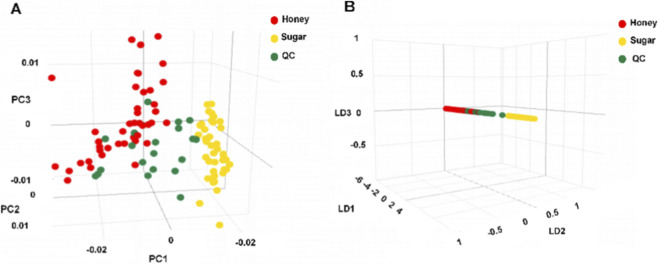
Multivariate analysis. **(A)** Tridimensional visualization of PCA. **(B)** Tridimensional visualization of PCA-LDA.

There were 110 REIMS profiles obtained from model samples, including 6 from HAR and 5 from SSAR. The chemometric model was processed and established using LiveID software. As an unsupervised dimensionality reduction algorithm, PCA maximizes the variance of data sets by ignoring class labels and identifying principal components. The PCA score plot (Figure 2A) revealed that heated honey processed products, heated soft sugar processed products, and QC samples were tightly clustered, respectively. This finding suggests that the analytical strategy was stable throughout a batch. Marked differences were detected between heated honey processed products and heated soft sugar processed products, suggesting differences in their metabolite characteristics. Linear discriminant analysis (LDA) is a supervised data analysis technique that maximizes the separation of multiple categories while minimizing within-category variation. The combination of unsupervised PCA with supervised LDA lowers the risk of overfitting in pure LDA models. Therefore, we employed a PCA-LDA model for multivariate statistical analysis to differentiate processed samples based on their molecular fingerprint. Figure 2B presents a three-dimensional PCA-LDA scatter plot, in which heated honey is completely discernible from heated soft sugar processed products.

### 3.2 REIMS real-time identification

Each sample was burnt by the iKnife and repeatedly cut 10 times. The relative standard deviation (RSD) of the intensity of 10 chromatographic peaks in each sample was <15%, suggesting the favorable repeatability and high accuracy of the obtained data. The identification model is based on typical REIMS spectral fingerprints. Using LiveID software, the real-time identification model rapidly identified samples online and visually displayed the identification results. Since the identification process was based on multi-objective factor fingerprints, the software evaluated the matching rate and provided a final identification.

**FIGURE 3:**
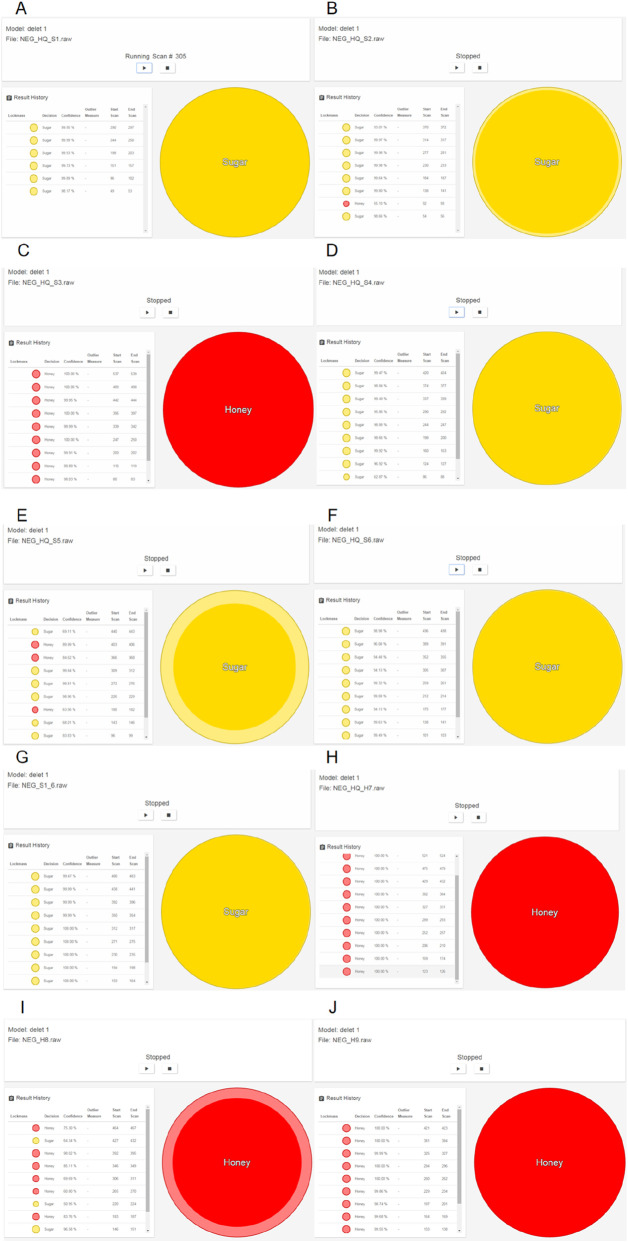
Real-time identification of the samples by LiveID of REIMS. **(A)** Real-time identification of S1 (sugar-adulterated). **(B)** Real-time identification of S2 (sugar-adulterated). **(C)** Real-time identification of S3 (HAR). **(D)** Real-time identification of S4 (sugar-adulterated). **(E)** Real-time identification of S5 (sugar-adulterated). **(F)** Real-time identification of S6 (sugar-adulterated). **(G)** Real-time identification of S_1-6_ (sugar-adulterated). **(H)** Real-time identification of H7 (HAR). **(I)** Real-time identification of H8 (HAR). **(J)** Real-time identification of H9 (HAR).

**FIGURE 4:**
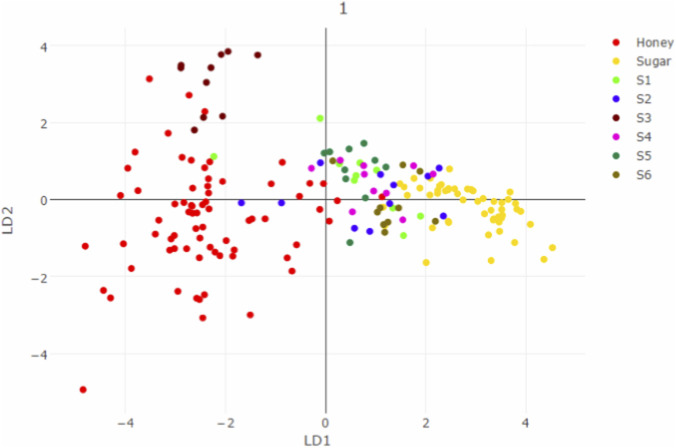
Distribution regions of AR processed with different proportions of heated sugar.

Real-time identification results for 10 samples revealed that S_1–6_, S1, S2, S4, S5, and S6 were SSAR, whereas S3, H7, H8, and H9 were HAR (Figure 3). Sample S2 was processed with heated granulated white sugar, sample S4 was processed with heated honey and heated soft sugar (1:1), sample S5 was processed with heated honey and heated granulated white sugar (1:1), and sample S6 was processed with heated soft sugar and heated granulated white sugar (1:1). All these samples were successfully identified as SSAR. This indicates that during HAR preparation, the use of soft or granulated white sugar instead of exclusively heated honey, or an admixture of the two and heated honey, will be identified as sugar-adulterated. PCA results for S1, S2, S3, S4, S5, and S6 revealed that AR processed with heated honey or heated sugar clustered into separate groups. HAR samples occupied quadrants 2 and 4, and heated-sugar processed AR occupied quadrants 1 and 3 (Figure 4). Of the samples identified as HAR, S3 and H7 were purchased from medical institutions, and H8 and H9 were processed with heated potpourri and linden honey, respectively. However, during the modeling process, heated locust flower honey was used to process AR. Therefore, AR processed with common heated honey varieties can be accurately identified as heated honey processed products, regardless of the honey variety. Accordingly, the model has the potential to accurately identify HAR in the market.

### 3.3 REIMS identification of AR processed with heated honey or sugar

**FIGURE 5:**
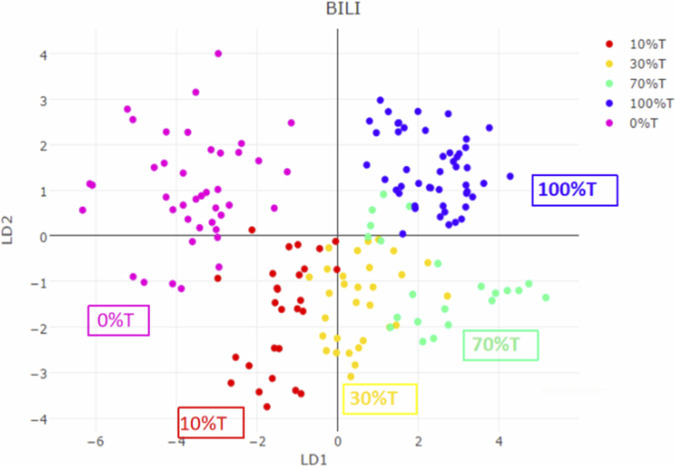
The identification of AR processed with different proportions of heated sugar.

We prepared heated honey samples mixed with 0%, 10%, 30%, 70%, and 100% heated soft sugar processed AR, with three batches for each proportion. From each batch of samples, 10 MS datasets were collected. When mixed with different proportions of heated soft sugar, PCA could still differentiate samples (Figure 5). Therefore, the model can identify HAR adulterated with soft or granulated white sugar and also predict the proportion of adulterated sugar.

**FIGURE 6:**
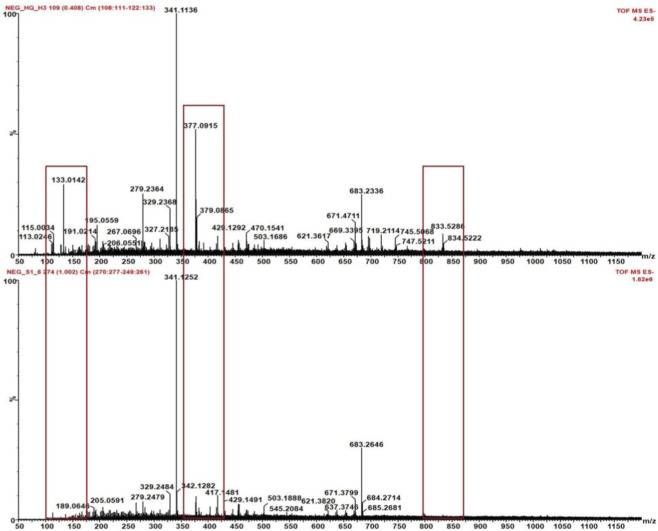
Differences in ions between HAR and heated sugar processed AR.

AR processed with heated honey can result in quantitative or/and qualitative changes in some metabolites, changing its medicinal effects. However, differences were also detected in ions between HAR and heated sugar processed AR (Figure 6), such as *m/z* 133.0142, m*/z* 377.0915, and *m/z* 833.5286. However, whether these differences can be applied to differentiate HAR from SSAR necessitates further studies.

### 3.4 Improvements in weight and spleen and thymus indexes

After 15 days of model establishment, rats in the model group exhibited symptoms, such as dark hair, lethargy, hyperactivity, and loose stool. Compared to the normal group, body weight and serum D-xylose levels were decreased in the experimental group (*P* < 0.01; Table 2); therefore, therefore, the presence of spleen deficiency was confirmed. In addition, the spleen and thymus indices were decreased in the model group.

**TABLE 2:** Weight and D-xylose level in rats with spleen deficiency.

Group name	Weight (g)	Rectal temperature (°C)	D-xylose (mg·mL-1)
NC	235.49 ± 11.51	37.15 ± 0.61	1.23 ± 0.12
MC	164.56 ± 21.46**	34.13 ± 0.77**	0.91 ± 0.04**

**P < 0.001, significantly different from NC.

**FIGURE 7:**
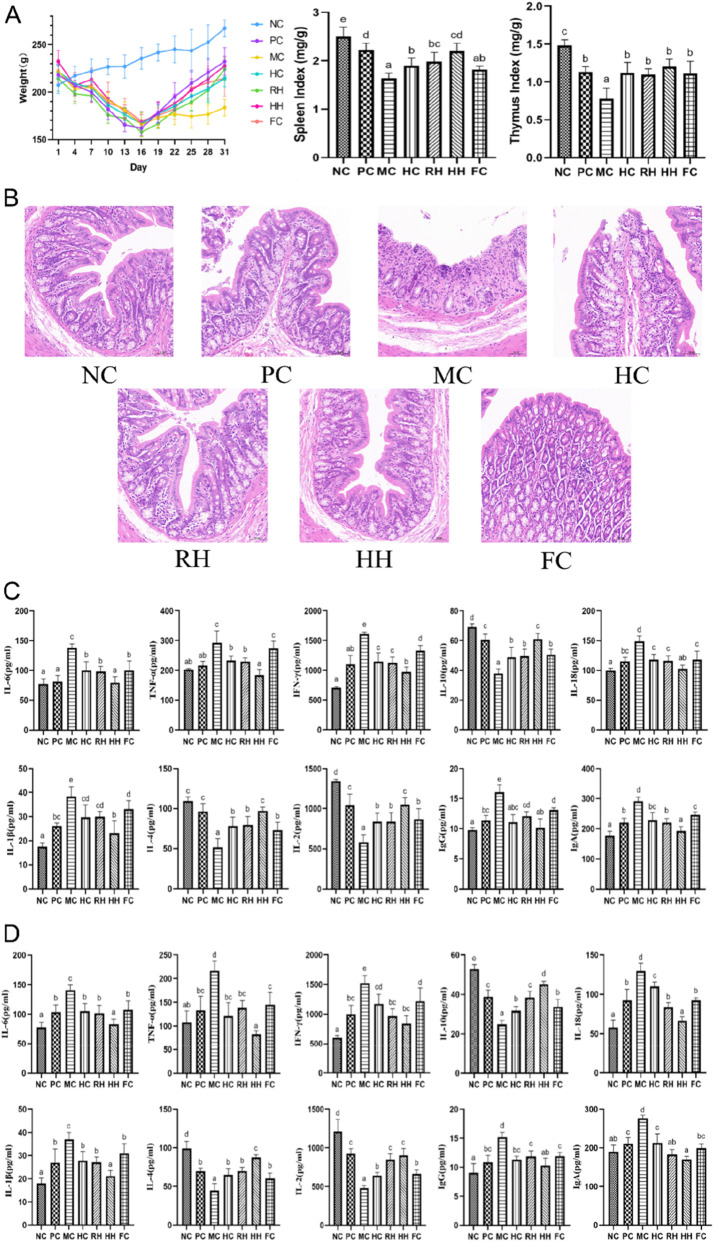
The effects of AR and HAR on routine indices, intestinal histopathological changes, and immune factor levels. **(A)** The effect of AR and HAR on body weight, spleen index, and thymus index (n = 6). **(B)** The effect of AR and HAR on colonic tissues (n = 3). **(C)** The effect of AR and HAR on the serum levels of immune factors (n = 6). **(D)** The effect of AR and HAR on the expression levels of immune factors in the colon (n = 6). a–d Different letters indicate significant difference at *P* < 0.05. All data are presented as mean ± SD.

The symptoms, such as dark hair, lethargy, hyperactivity, and loose stools, improved in all groups of modeling rats. Body weight, spleen index, and thymus index significantly increased after administering different drugs, but there was no significant difference between AR and HAR in this regard (Figure 7A).

### 3.5 H&E staining

Intestinal tissue structure was normal in the NC group. Epithelial cells of the mucosal layer were extensively detached in the MC group, exposing the lamina propria. The number of crypts was significantly decreased, and some crypts were missing in this group. The submucosal layer exhibited mild edema, connective tissue hyperplasia, enlarged interstitial spaces, and inflammatory cell infiltration. In the PC group, epithelial cells in the mucosal layer were tightly arranged without detachment, or obvious inflammatory cell infiltration. In the HC group, some epithelial cells of the mucosal layer were detached, the lamina propria was exposed, the crypts were structurally intact and neatly arranged, with no sign of inflammatory cell infiltration. In the RH group, epithelial cells were tightly arranged in the mucosal layer without detachment, the number of crypts was decreased, and the crypts were sparsely arranged. Mild inflammatory cell infiltration was observed in the RH group. The HH group exhibited well-preserved mucosal architecture, characterized by normal epithelial stratification without desquamation. There were structurally integrated crypts with well-organized glandular patterns (crypt density: 48.6 ± 3.2/mm^2^ vs. 32.1 ± 2.8 in controls, *P* < 0.05). There was no obvious sign of inflammatory cell infiltration. In the FC group, epithelial cells were tightly arranged in the mucosal layer without detachment, crypts were structurally intact and neatly arranged, with mild inflammatory cell infiltration (Figure 7B).

### 3.6 The levels of immune factors in the serum and colon samples

Compared to the normal control group (NC), pro-inflammatory factors (IL-6, IFN-γ, IL-18, IL-1β, and TNF-α) and immunoglobulins (IgA and IgG) were significantly upregulated in the serum sample and colon tissues of the experimental group (*P* < 0.05), while anti-inflammatory factors, including IL-10, IL-4, and T-cell regulatory factor IL-2 were significantly downregulated in this group (*P* < 0.05). Compared to the MC group, both AR and HAR significantly modulated IL-6, IFN-γ, IL-18, IL-1β, TNF-α, IgA, IgG, IL-10, IL-4, and IL-2 levels in the serum and colon samples (*P* < 0.05). The regulatory effect of HAR was superior to that of AR, but there was no significant difference between AR and HAR in regulating IFN-γ, IL-2, and IgA levels in the colon. Meanwhile, HC and FC significantly modulated the expression levels of the above-mentioned immune factors in serum and colon samples (*P* < 0.05), and their regulatory effects were similar to those of AR (Figures 7C,D).

### 3.7 Changes in the richness and diversity of intestinal flora after treatment with AR and HAR

**FIGURE 8:**
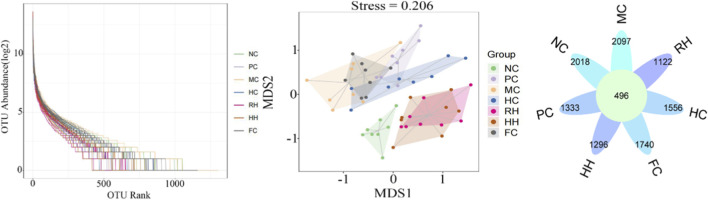
Alpha and beta diversity analysis.

Alpha diversity analysis based on OTU level exhibited that the rank-abundance curves of each group decreased smoothly, the horizontal axis became wider, and the Simpson’s index was close to 0.60. These findings indicate that the diversity of each group of colonies was rich, the composition of species was homogeneous, and the sequencing depth was reasonable. Regarding beta diversity, principal coordinates analysis (PCoA) revealed that the NC and MC groups were independently clustered with a certain distance, suggesting significant differences in the composition of their flora. After treatment with different administration components, HC, RH, and HH modulate the intestinal flora, making it more similar to the NC group. RH and HH exhibited marked regulatory effect, and HH was superior to RH. As shown by the flower diagram, the NC, MC, PC, HC, RH, HH, and FC groups possessed 2514, 2593, 1829, 2052, 1618, 1792, and 2236 OTUs, respectively. The total number of OTUs was 496 in the 7 groups. The NC, MC, PC, HC, RH, HH, and FC groups possessed 2018, 2097, 1333, 1556, 1122, 1296, and 1740 OTUs, respectively (Figure 8).

**FIGURE 9:**
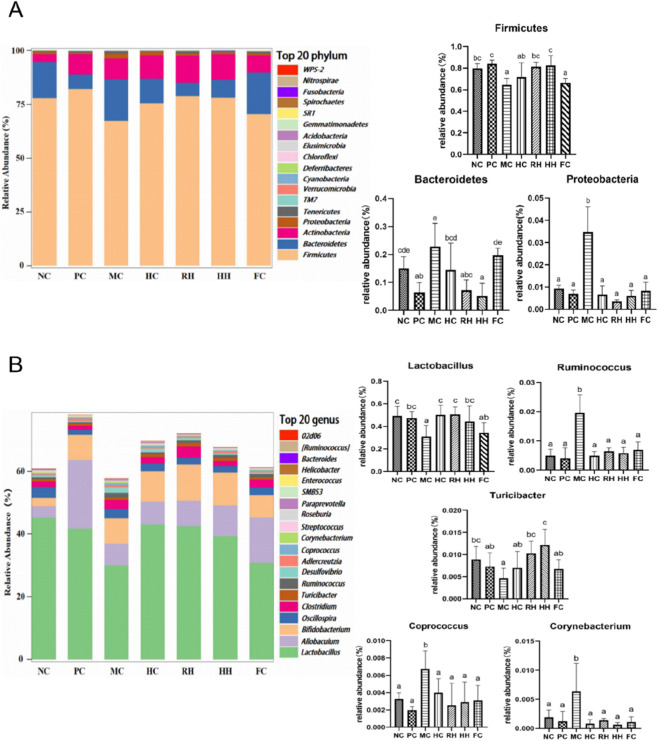
The regulatory effects of AR and HAR on intestinal flora. **(A)** Phylum level. **(B)** Genus level. a–d Different letters indicate significant difference at *P* < 0.05. All data are presented as mean ± SD (n = 8).

In the NC group, gut microbiota were mainly composed of *Firmicutes*, *Bacteroidetes,* and *Proteobacteria* at the phylum level. At the genus level, gut microbiota mainly included *Lactobacillus*, *Oscillospira*, *Ruminococcus*, *Turicibacter*, *Desulfovibrio*, *Coprococcus*, and *Corynebacterium*. Compared to the NC group, the abundance of *Firmicutes* was significantly lower in the MC group (*P* < 0.05) and the abundance of *Proteobacteria* was significantly higher in the MC group (*P* < 0.05). The abundance of *Bacteroidetes* was also higher in the MC group, but the difference was not statistically significant (*P* > 0.05). At the genus level, the abundance of *Lactobacillus* and *Turicibacter* was significantly lower in the MC group (*P* < 0.05), and the abundance of *Ruminococcus*, *Coprococcus*, and *Corynebacterium* was significantly higher in this group (*P* < 0.05). Compared to the model group, all drugs significantly increased the abundance of *Firmicutes* (*P* < 0.05); however, the regulatory effect of HC did not meet the threshold of statistical significance. Meanwhile, all drugs significantly decreased the abundance of *Bacteroidetes* and *Proteobacteria* (*P* < 0.05). The abundance of *Lactobacillus* was significantly increased in the HC, RH, and HH groups (*P* < 0.05). The abundance of *Ruminococcus*, *Coprococcus,* and *Corynebacterium* was significantly decreased in all groups (*P* < 0.05), and the abundance of *Turicibacter* was significantly increased in the RH and HH groups (*P* < 0.05) (Figures 9A,B).

### 3.8 Changes in the fecal content of SCFAs after treatment with AR and HAR

**FIGURE 10:**
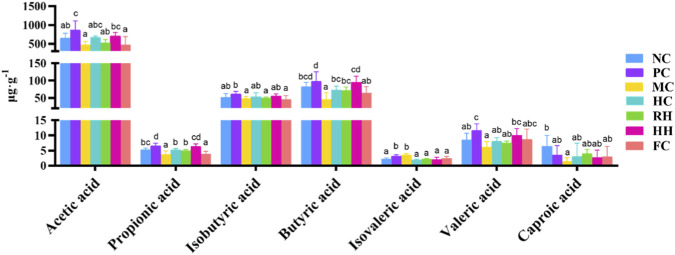
The regulatory effects of AR and HAR on the concentration of SCFAs. a–d Different letters indicate significant difference at *P* < 0.05. All data are presented as mean ± SD (n = 8).

Both AR and HAR significantly increased the concentrations of propionic acid and butyric acid and decreased the concentrations of isovaleric acid in the colon of rats with spleen deficiency (Figure 10). Although they did not affect the tissue concentrations of acetic acid, isobutyric acid, valeric acid, and capric acid. The effect of HAR on propionic acid production was greater than that of AR.

### 3.9 Correlation analysis

#### 3.9.1 Correlation analysis between immune factors and intestinal flora at the genus level

**FIGURE 11:**
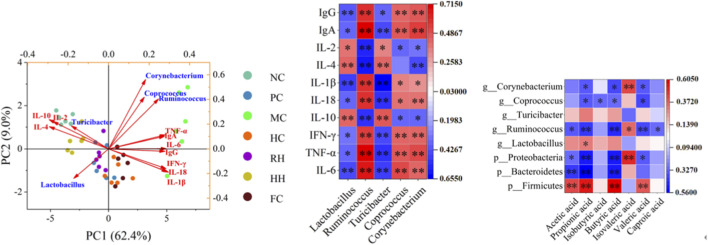
Correlation analysis between intestinal flora, immune factors, and SCFAs. All data are presented as mean ± SD (n = 6), **P < 0.05, *P < 0.01.

There was a correlation between changes in the abundance of homoeopathogenic bacteria and pro-inflammatory factors levels in rats with spleen deficiency (Figure 11). The beneficial effects of HAR on spleen deficiency were correlated with the abundance of beneficial bacteria and anti-inflammatory factor levels.

IgG, IgA, IL-1β, IL-18, IFN-γ, TNF-α, and IL-6 levels were negatively correlated with the abundance of *Lactobacillus* (*P* < 0.05 or *P* < 0.01, blue). IL-2, IL-4, and IL-10 levels were positively correlated with the abundance of *Lactobacillus* (*P* < 0.01, red). IL-2, IL-4, and IL-10 levels were negatively correlated with the abundance of *Ruminococcus* (*P* < 0.01, blue). IgG, IgA, IL-1β, IL-18, IFN-γ, TNF-α, and IL-6 levels were positively correlated with the abundance of *Ruminococcus* (*P* < 0.05, red). IgG, IgA, IL-1β, IL-18, IFN-γ, TNF-α, and IL-6 levels were negatively correlated with the abundance of *Turicibacter* (*P* < 0.05 or *P* < 0.01, blue). IL-2, IL-4, and IL-10 levels were positively correlated with the abundance of *Turicibacter* (*P* < 0.05 or *P* < 0.01, red). IgG, IgA, IL-1β, IL-18, IFN-γ, TNF-α, and IL-6 levels were positively correlated with the abundance of *Coprococcus* (*P* < 0.05 or *P* < 0.01, red). IL-2 and IL-10 levels were negatively correlated with the abundance of *Coprococcus* (*P* < 0.05, blue). IgG, IgA, IL-1β, IL-18, IFN-γ, TNF-α, and IL-6 levels were positively correlated with the abundance of *Corynebacterium* (*P* < 0.05 or *P* < 0.01, red). IL-2, IL-4, and IL-10 levels were negatively correlated with the abundance of *Corynebacterium* (*P* < 0.05 or *P* < 0.01, blue).

#### 3.9.2 Association between SCFAs and gut microbiota

At the genus level, the abundance of *Corynebacterium* was negatively correlated with the concentration of propionic acid, butyric acid, and valeric acid (blue, *P* < 0.01) and positively correlated with the concentration of isovaleric acid (red, *P* < 0.05). The abundance of *Coprococcus* was negatively correlated with propionic acid, isobutyric acid, butyric acid, and valeric acid levels (blue, *P* < 0.05). The abundance of *Ruminococcus* was negatively correlated with acetic acid, propionic acid, butyric acid, valeric acid, and caproic acid concentrations (blue, *P* < 0.05 or *P* < 0.01) and positively correlated with isovaleric acid concentrations (red, *P* < 0.05). The abundance of *Lactobacillus* was positively correlated with propionic acid concentrations (red, *P* < 0.05). The abundance of *Proteobacteria* was negatively correlated with acetic acid, propionic acid, butyric acid, and valeric acid concentrations (blue, *P* < 0.05 or *P* < 0.01) and positively correlated with isovaleric acid concentrations (red, *P* < 0.01). The abundance of *Bacteroidetes* was negatively correlated with acetic acid, propionic acid, and butyric acid concentrations (blue, *P* < 0.01). The abundance of *Firmicutes* was positively correlated with the concentration of acetic acid, propionic acid, butyric acid, and valeric acid (red, *P* < 0.01).

## 4 Discussion

The REIMS used in this study requires simple sample pretreatment and can provide detection results within 2 s after sample preparation, enabling *in-situ*, real-time, and high-throughput analysis of samples. This study innovatively applied REIMS to TCM processing. This advanced MS technique provides a rapid and reliable method for identifying differences between raw and processed Chinese medicinal decoction pieces, as well as for their quality evaluation.

According to the TCM theory, the spleen and stomach are the root of acquired constitution, governing the transportation and transformation of food essence, and serving as the source of qi and blood production. Weakness of the spleen and stomach impairs transportation and transformation, and lead to indigestion, internal accumulation of dampness, and downward flow of turbid fluid mixed with clear fluid, manifesting as diarrhea. From the perspective of immune activity, intestinal flora, and short-chain fatty acid metabolism, this study investigated the ameliorative effects of AR on rats with spleen deficiency before and after heated honey processing, preliminarily revealing the mechanism underlying the enhanced efficacy of HAR.

Direct drug targets are the source of pharmacological effects, representing the most direct, critical, and initial biological basis behind disease treatment. Recently, with the rapid development of frontier disciplines such as molecular biology, chemical biology, and biophysics, researchers have explored and established a series of methods for the identification and recognition of direct drug targets. These methods have been applied to investigate direct targets of metabolites and compound prescriptions of TCM, yielding numerous breakthrough achievements (Zhang et al., 2022; Liu et al., 2022; Zhou et al., 2022). Nevertheless, there are few studies on the direct targets of HAR in ameliorating spleen deficiency. Therefore, target-fishing technology will be adopted in future studies to explore the direct target proteins of HAR in spleen deficiency. This will help clarify the scientific connotation of HAR at the target source, thereby providing a reference for the rational clinical application of HAR.

## 5 Conclusion

A real-time differential identification analysis model based on REIMS technology was developed to rapidly and accurately identify if the processing of AR product has been adulterated with sugar. This technique provides new ideas and approaches for authenticity identification, species differentiation, and quality evaluation of TCMs.

Spleen deficiency can lead to gut dysbiosis in rats, reduce the abundance of beneficial bacteria, and increase the abundance of pathogenic bacteria, thereby affecting the production of SCFAs and inflammatory factors and resulting in intestinal diseases, such as diarrhea. Treatment with AR and HAR ameliorated gut dysbiosis, increased the abundance of beneficial bacteria, and reduced the abundance of pathogenic bacteria. AR and HAR also promoted the synthesis of propionic acid and butyric acid, reinforced the metabolism of isovaleric acid, and enhanced the specific and non-specific immune responses of rats, thereby improving diarrhea in rats with spleen deficiency. In particular, the significant ameliorative effects of HAR on splenic deficiency in rats were associated with an increase in the abundance of *Lactobacillus* and *Turicibacter*, increased levels of propionic acid in the intestinal tract of rats, upregulation of anti-inflammatory factors, and downregulation of pro-inflammatory factors. This can provide a reference basis for explaining the enhanced effect of replenishing qi and tonifying the middle-jiao of HAR.

## Data Availability

The original contributions presented in the study are publicly available. This data can be found here: https://doi.org/10.6084/m9.figshare.32086956.
