# Supplementary material for: A novel method for real-time adulteration identification of heated-honey processed Astragali radix using REIMS-iKnife and its regulatory effects on gut microbiota, SCFAs, and immune function in spleen-deficient rats
Source: Front Pharmacol. 2026 Apr 29;17:1711584. doi: 10.3389/fphar.2026.1711584 (PMC13168081; doi:10.3389/fphar.2026.1711584)
Supplement: Supplementary file 1 [file Image1.pdf]

Figure S1

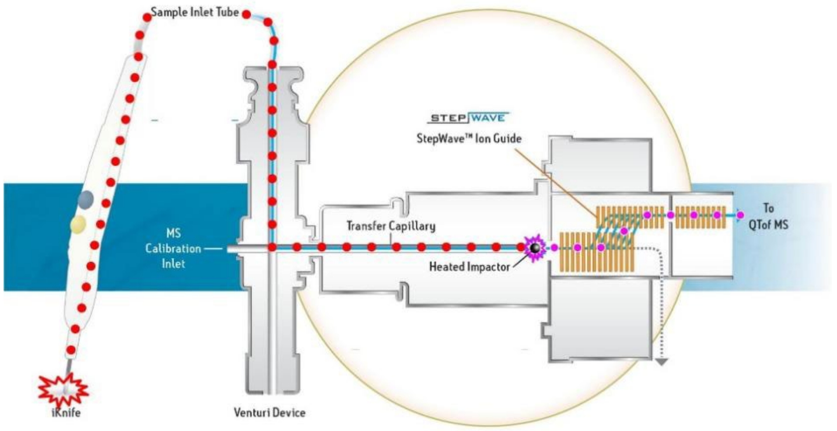

REIMS experimental setup employed for sampling using a high current electrosurgical knife (iKnife).
